# Advanced Competencies for Endoscopy Nurses: A Rapid Review of Current Practices and Training Approaches

**DOI:** 10.1111/nhs.70328

**Published:** 2026-04-05

**Authors:** Andrea Minciullo, Daniela Tartaglini, Lucia Filomeno, Riccardo Grande, Dhurata Ivziku

**Affiliations:** ^1^ Department of Biomedicine and Prevention Tor Vergata University of Rome Rome Italy; ^2^ Department of Health Professions Fondazione Policlinico Universitario Campus Bio‐Medico Rome Italy; ^3^ Department of Clinical and Molecular Medicine Sapienza University of Rome Rome Italy; ^4^ Gastroenterology and Endoscopy Unit Fondazione Policlinico Universitario Campus Bio‐Medico Rome Italy

**Keywords:** advanced competencies, endoscopy, literature review, nurse

## Abstract

As endoscopy procedures expand in scope and technology, endoscopy nurses find themselves requiring advanced competencies that span both technical skills and clinical knowledge. This review aims to identify the key advanced competencies for nurses in endoscopy, addressing competencies that optimize patient outcomes and the efficacy of endoscopic procedures. This review was conducted in accordance with Cochrane Rapid Review guidance between June and September 2025 using MeSH terms including “*endoscopy*,” “*gastrointestinal endoscopy*,” “*nurse*,” “*nursing*,” “*competence*,” “*competency*,” “*advanced practice*,” and “*training*.” PubMed, CINAHL and Scopus were searched for peer‐reviewed articles and gray literature, with no language or time limitations. Sixteen articles were included. The core competency domains identified are: clinical assessment and patient management, procedural and interventional support competence, equipment management and infection control, sedation and patient monitoring, documentation and quality assurance. This rapid review provides an updated synthesis of advanced competencies in gastrointestinal endoscopy nursing. While core domains are recognized internationally, variation in training structures and role delineation persists. Findings contribute to the development of harmonized frameworks and inform future curriculum design and workforce planning.

## Keypoints

- The review identifies six core domains of advanced competencies for endoscopy nurses: clinical assessment and patient management, procedural and interventional support competence, equipment management and infection control, sedation and patient monitoring, documentation, and quality assurance.
- Structured competency frameworks and standardized training programs are essential to ensure consistency, enhance patient safety, and promote professional development in gastrointestinal endoscopy nursing.
- The global evolution of endoscopy nursing toward advanced and autonomous practice highlights the need for continuous education, technological adaptation, and formal recognition of specialized nursing roles.

## 1 Introduction

In recent years, the role of nursing in endoscopy has evolved substantially, driven by the increasing complexity of modern healthcare and rising expectations for quality assurance in procedural environments (Yu and Roh 2018; Dunkley et al. 2019). Gastrointestinal endoscopy demands not only advanced technical skills but also comprehensive knowledge of patient care, procedural safety, and effective collaboration within multidisciplinary teams (Yu and Roh 2018; Li et al. 2024; Schoefl 2023).

Despite the growing body of literature describing endoscopy nursing roles, there remains limited synthesis of advanced competencies across countries, training systems, and regulatory contexts. Existing publications often focus on single‐national experiences, specific procedures, or conceptual discussions of advanced practice, without providing an updated cross‐regional mapping of competency domains and training approaches (Notarnicola et al. 2023).

This rapid review addresses that gap by providing an updated, cross‐regional synthesis of advanced competencies and training approaches for nurses working in gastrointestinal endoscopy. By moving beyond descriptive accounts of evolving roles, the review aims to support harmonization of competency frameworks and inform curriculum development and workforce planning.

Integrating advanced competencies into endoscopy nursing practice is essential for fostering the holistic development of the profession. Educational programs designed to address the specific challenges of gastrointestinal endoscopy must adapt to an evolving landscape characterized by rapid technological innovation and new procedural techniques (Li et al. 2024; Dunkley et al. 2019). Such programs emphasize the importance of continuous professional development, ensuring that nurses remain agile in the face of technological change while consistently delivering high‐quality care (Dunkley et al. 2019; Parrella et al. 2025).

The Italian national survey conducted by ANOTE (Associazione Nazionale Operatori Tecniche Endoscopiche)—ANIGEA (Associazione Nazionale Infermieri di Gastroenterologia e Associati) further illustrates this dynamic context, revealing significant organizational, educational, and structural challenges within digestive endoscopy nursing (Guarini et al. 2018). In Italy, endoscopy nurses are pivotal in providing comprehensive gastroenterological care across outpatient clinics, diagnostic and therapeutic units, and inpatient wards; yet the findings highlight substantial disparities that underscore the need for standardized training, infrastructure enhancement, and role recognition.

An expanding body of research highlights the critical importance of competency assessment frameworks to ensure that endoscopy nurses are fully equipped to manage the complex procedures inherent to their practice (Siau et al. 2019; Li et al. 2024). Recent developments in competency evaluation methods and training platforms, such as JETS (Joint Endoscopy Training System) Workforce, offer frameworks for documenting and assessing the abilities of endoscopy nurses (Siau et al. 2019; Munnelly et al. 2021). These platforms assist in professional revalidation and serve to pinpoint areas where further training may be required, aligning nursing competencies with best practices in procedural care (Embertson et al. 2020; Munnelly et al. 2021). Such systematic approaches are imperative for cultivating a workforce adept in managing both basic and advanced endoscopy practices, thereby ensuring a competent nursing presence in this specialty.

The JETS Workforce model represents a significant advancement in the ongoing professional development of nurses in the endoscopy field, specifically catering to their competency management and educational needs. This e‐portfolio platform was developed in response to calls for an effective means to document and assess the competencies of endoscopy nurses, thus facilitating their revalidation processes (Siau et al. 2019). The JETS Workforce is designed to support nurses in tracking their learning and performance, ensuring they maintain the requisite skills to deliver safe and high‐quality patient care during endoscopic procedures. By integrating comprehensive competence assessments into training programs, the JETS model reinforces the need for ongoing education and feedback mechanisms, allowing nurses to measure their progress against predefined professional standards (Yu and Roh 2018).

Prahalad defined the notion of “core competence” as a form of collective learning characterized by the adeptness to synchronize diverse production skills and integrate various processes into a cohesive whole. This perspective underscores the intricate web of interactions among individuals, their competencies, available resources, and the contextual frameworks in which they operate (Prahalad and Hamel 2007). To adequately assess the extent to which individuals advance in developing these competencies, various evaluation tools have been established.

Also, McClelland posits that while competencies are inherently centered around the individual, they cannot be entirely divorced from the context within which they manifest; thus, competencies are not solely attributes of the individual but are also deeply influenced by their specific environmental setting (McClelland 1976). In this regard, Patricia Benner has articulated a framework (Benner 1984) delineating the stages of clinical competence that nurses traverse throughout their professional journey, categorizing these stages into five primary levels: (1) Novice, (2) Advanced Beginner, (3) Competent, (4) Proficient, and (5) Expert. This framework posits that for knowledge to evolve significantly, it should be rooted in both practical and clinical learning experiences, which ultimately enable practitioners to interweave theoretical understanding with real‐life application (Field 2004). A nurse who embarks on providing endoscopy services for the first time typically commences at the Novice level, progressively advancing through the stages until reaching the Expert level, as they accumulate both experience and proficiency.

The literature on nurse endoscopists' advanced competencies reveals significant gaps, particularly concerning standardized training frameworks and competency assessment tools. While studies have highlighted the safety and effectiveness of nurse‐led endoscopic procedures, there is a lack of universally accepted competency evaluation systems. For instance, a recent Delphi study in China developed a competency evaluation index for nurse endoscopists at various stages, but such models are not widely implemented or validated internationally (Fang et al. 2025). This heterogeneity creates uncertainty regarding core domains of practice and limits the transferability of training models across settings.

Furthermore, there is limited research on the integration of advanced endoscopic techniques into nursing curricula and the long‐term outcomes of nurse‐led procedures. A systematic review noted the absence of standardized training programs for nurse endoscopists, which hinders the development of comprehensive educational strategies (Sprout 2000).

In summary, the critical evaluation of advanced nursing competencies within the endoscopy context is essential for promoting best practices and enhancing patient safety. Previous published reviews provided valuable insights into endoscopy nursing roles but were limited in scope. They either focused on single‐country contexts, described roles without mapping them to structured training frameworks, or highlighted advanced practice only conceptually. None offered a timely synthesis that integrates international evidence, emerging technologies, and training innovations. This rapid review responds to that gap by delivering an up‐to‐date, cross‐regional appraisal of advanced competencies, training approaches, and competency assessment frameworks, thereby supporting the development of standardized and future‐oriented curricula for endoscopy nurses.

By examining educational trends, competency assessments, and the implications of evolving technologies, this review aims to contribute significantly to the body of knowledge surrounding nursing practices in endoscopy, ultimately fostering better patient care outcomes. Therefore, this review aims to identify and summarize the key advanced competencies required for nurses in endoscopy, addressing both the expected knowledge and skill sets that optimize patient outcomes and enhance the efficacy of endoscopic procedures.

## 2 Methods

This rapid review employed a systematic approach to identify, synthesize, and critically appraise existing literature on advanced competencies for nurses working in the endoscopy setting. The methodology adhered to established rapid review guidelines, including those outlined by the Cochrane Rapid Review Methods Group, which emphasize the need for flexibility and pragmatic approaches to respond to urgent healthcare questions efficiently (King et al. 2022; Tricco et al. 2022).

### 2.1 Search Strategy

A comprehensive literature search was conducted using multiple electronic databases, including PubMed, CINAHL, and Scopus. The search strategy was designed to capture relevant studies by combining keywords and medical subject headings (MeSH) related to “endoscopy,” “nursing competencies,” “professional development,” and “training.” The search embraced peer‐reviewed and not peer‐reviewed articles, gray literature, position statements and guidelines with no time or language limitations, ensuring the inclusion of recent advancements in nurse training and competency models (Mijumbi‐Deve et al. 2022).

The search was performed between June and September 2025 and covered all records from database inception.

Search terms were developed through preliminary scoping and combined controlled vocabulary (e.g., MeSH in PubMed) and free‐text keywords. The core search string used in PubMed was as follows:

(“*Endoscopy*”*[MeSH] OR* “*Gastrointestinal Endoscopy*” *OR endoscop*) *AND* (“*Nurses*”*[MeSH] OR nurse* OR “endoscopy nurse*” OR “gastroenterology nurse*”) AND (competenc* OR “core competenc*” OR “advanced practice” OR “professional role*” OR training OR education OR curriculum).

Equivalent keyword combinations and Boolean operators were adapted for CINAHL and Scopus according to database‐specific indexing systems. Boolean operators (AND/OR) were applied systematically, and truncation (*) was used where appropriate to capture term variations.

### 2.2 Study Selection

Eligibility criteria were established to focus on studies that specifically addressed competencies in endoscopy nursing, including educational programs, training frameworks, and competency assessments. Studies that did not directly investigate nursing competencies in the context of endoscopy were excluded. Two independent reviewers screened titles and abstracts for relevance, followed by a full‐text review of the remaining articles.

Before screening commenced, the reviewers piloted the eligibility criteria on a random sample of 15 records to ensure shared understanding and consistent application of inclusion and exclusion criteria. Minor clarifications to the eligibility definitions were made at this stage.

Title and abstract screening was performed independently by both reviewers. Records considered potentially eligible by at least one reviewer proceeded to full‐text assessment.

Full‐text screening was also conducted independently by two reviewers using a structured eligibility checklist. Reasons for exclusion at the full‐text stage were documented (e.g., no relevant nursing population, no competency focus, unavailable full text).

Discrepancies were resolved through discussion and consensus to enhance the reliability of study selection (Spry and Mierzwinski‐Urban 2018; Moore et al. 2018).

If consensus could not be reached through discussion, a third senior reviewer was available for arbitration; however, no arbitration was ultimately required.

A PRISMA flow diagram was used to document the study selection process, including numbers of records identified, screened, excluded (with reasons), and included in the final synthesis.

### 2.3 Quality Appraisal

Given the heterogeneity of included sources—comprising qualitative studies, mixed‐method research, developmental frameworks, consensus statements, and professional guidelines—a single standardized risk‐of‐bias tool was not appropriate for all study types. Nevertheless, methodological quality was not omitted.

For empirical research studies (qualitative, mixed‐method, and developmental designs), methodological quality was assessed using the appropriate Joanna Briggs Institute (JBI) critical appraisal tools.

Guidelines, consensus documents, and position statements were not subjected to formal risk‐of‐bias scoring, as these documents aim to provide professional standards rather than generate primary empirical evidence.

### 2.4 Data Extraction and Synthesis

Data were extracted using a standardized form that included information on study design, population characteristics, training programs, competency frameworks, and outcomes.

A structured data extraction form was specifically developed for this review prior to full extraction. The form was piloted on three included studies and refined to ensure clarity and consistency.

The extraction template included the following predefined fields: (1) bibliographic details (author, year, country); (2) study design or document type; (3) setting and population; (4) stated objectives; (5) description of competency domains; (6) description of training or assessment frameworks; (7) reported outcomes or implications for practice; (8) notes on methodological characteristics (where applicable).

Key themes were derived through a thematic analysis approach, allowing for the identification of common competencies highlighted across studies (Garritty et al. 2024).

An inductive thematic synthesis was performed. Extracted competency‐related data were coded line‐by‐line, grouped into descriptive categories, and subsequently organized into higher‐order domains. The final competency domains were agreed upon through iterative discussion among the research team to ensure conceptual coherence and transparency.

A narrative synthesis was performed to organize the findings into coherent categories, reflecting the different dimensions of competencies necessary for endoscopy nursing practice (Xu et al. 2022).

### 2.5 Eligibility Criteria

Studies were selected according to predefined inclusion and exclusion criteria.

Inclusion criteria were as follows:

(1) Population:

Registered nurses, advanced practice nurses, specialist nurses, or nurse endoscopists working in gastrointestinal endoscopy settings.

(2) Concept:

Explicit description, definition, development, assessment, or evaluation of professional competencies, advanced roles, training programs, curricula, or competency frameworks related to endoscopy nursing.

(3) Context:

Gastrointestinal endoscopy units (diagnostic and/or therapeutic), including hospital, outpatient, or specialized endoscopy center settings.

(4) Types of sources:

Empirical research studies (qualitative, quantitative, mixed‐method), developmental studies, consensus statements (e.g., Delphi), professional guidelines, and position statements.

Exclusion criteria were as follows:

(1) Studies focusing exclusively on physicians or non‐nursing professionals without explicit reference to nursing competencies.

(2) Articles describing general nursing competencies not specific to gastrointestinal endoscopy.

(3) Clinical studies addressing procedural outcomes (e.g., complication rates, technical efficacy) without discussion of nursing roles, competencies, or training.

(4) Articles for which full text was not accessible.

Studies were required to contain an explicit reference to competency‐related constructs (e.g., “competence,” “competency,” “role delineation,” “advanced practice,” “training framework,” or equivalent terms) within the context of gastrointestinal endoscopy nursing in order to be considered eligible.

## 3 Results

**FIGURE 1:**
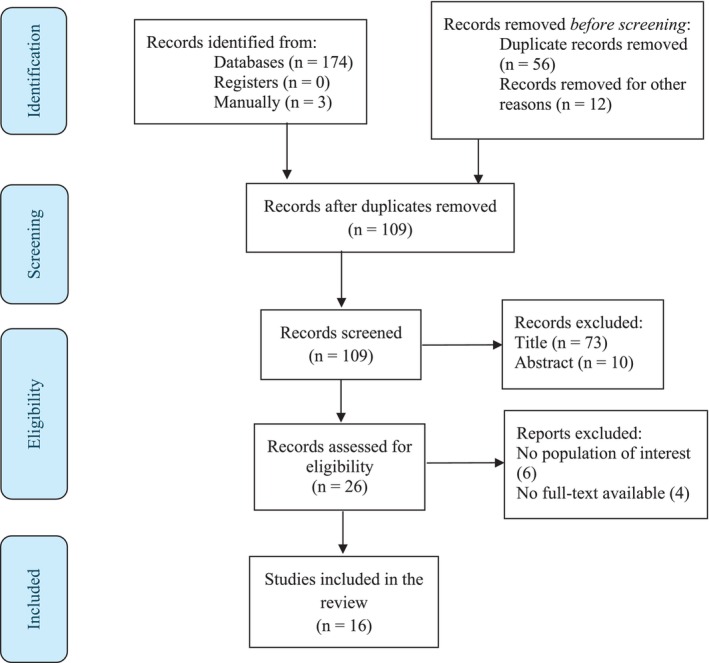
PRISMA flow diagram (Page et al. 2021).

A total of 177 articles returned from electronic databases were screened for inclusion. After adequate evaluation, 16 studies were included (Figure 1).

The findings from the literature review reveal significant insights into the competencies and roles of nurses in gastrointestinal endoscopy.

### 3.1 Study Characteristics

Sixteen sources were included (see Table 1), comprising developmental studies, qualitative and mixed‐method studies, literature and scoping reviews, expert guidelines, and position statements. These originated from diverse countries, including Europe (European Society of Gastroenterology and Endoscopy Nurses and Associates—ESGENA 2004), North America (Society of Gastroenterology Nurses and Associates—SGNA 2010; Society of Gastroenterology Nurses and Associates—SGNA 2013, 2014), Asia (Siriwat et al. 2020; Ren et al. 2019; Huang et al. 2021), and multiple European countries including Italy (Minciullo and Filomeno 2024; Napolitano et al. 2025; Parrella et al. 2025), Spain (Alouali Moussakhkhar et al. 2023; Gómez and Llach 2009), and Finland (Vuorinen et al. 2009). The primary objectives of these studies and guidelines were to define competencies, delineate roles, develop training curricula, and propose standards for gastrointestinal (GI) endoscopy nursing.

**TABLE 1:** Data extraction table.

References	Objective	Design/type and sample (if empirical)	Results
*Manipulation of endoscopes during endoscopic procedures by gastroenterology nurses and associates* SGNA Board of Directors (2010) USA	/	Position statement	Ability to manipulate the endoscope Understand complications, identify symptoms and initiate interventions Comprehensive care of the patient
*European Job Profile for Endoscopy Nurses—August 2004* European Society of Gastroenterology and Endoscopy Nurses and Associates (ESGENA) Europe	To offer clear and structured information about the endoscopy nursing; to promote discussion in the membership countries concerning endoscopy nurses as a recognized specialty.	Job profile in some European Countries.	Holistic patient care Technical assistance during diagnostic and therapeutic procedures Care of endoscopic equipment Hygiene and infection control and prevention, including reprocessing of endoscopic equipment Documentation and record‐keeping Organization and clinical management Health and safety Legal and ethical aspects Research Health and disease education of patient and carers Education and training of staff Quality control
*Ruolo dell'infermiere in ecoendoscopia* Salvetto (2007) Italy	To define the role of nurses in endoscopic ultrasonography.	Qualitative descriptive design	Management of instrumentation Knowledge of instruments Ability to handle complications Relationship with patient and family Monitoring of vital signs Disinfection, processing, and disposal of instruments Knowledge of anatomy Knowledge of ultrasound images Emergency management
*ESGENA Core Curriculum for Endoscopy Nursing* (European Society of Gastroenterology and Endoscopy Nurses and Associates) Beilenhoff et al. (2011) Europe	Create a European core curriculum for endoscopy nursing to harmonize training and professional standards across Europe.	Collaboration among national representatives, literature reviews, and consensus development meetings.	Comprehensive patient care Technical assistance during diagnostic and therapeutic procedures Care of endoscopic equipment Hygiene and infection control and prevention, including reprocessing of endoscopic equipment Documentation and record‐keeping Organization and clinical management Health and safety Legal and ethical aspects Research Providing patients and carers with information about health and disease Education and training of staff Quality control
*Papel de la enfermera en la endoscopia digestiva* Gómez and Llach (2009) Spain	To define the role of nurses in gastrointestinal endoscopy	Descriptive study design	Care competencies Educational competencies Research competencies Managerial competencies
*Endoscopy Nurse as a Patient Care Coordinator The Expanded Role of the Competent Nurse in the Gastroenterology Setting* Vuorinen et al. (2009) Finland	To describe the expanded roles of registered nurses as patient care coordinators and evaluate their performance in gastroenterology.	Qualitative study design.	Helping role Teaching‐coaching function Diagnostic and monitoring function Effective management of situations Administering and monitoring Monitoring and ensuring quality Organizational and work role
*Guidelines for Nursing Documentation in Gastrointestinal Endoscopy* SGNA—Society of Gastroenterology Nurses and Associates (2013) USA	To provide guidelines for institutional policies and practices related to nursing documentation in gastrointestinal endoscopy.	Expert committee guidelines development and literature‐based recommendations.	Cardiac monitoring Capnography Assessment of skin condition NPO status Bowel preparation Fall risk assessment Pregnancy status assessment Nutritional status assessment Abdominal assessment Height and weight measurement Activities of daily living assessment Addressing emotional and psychological needs
*Standards of Clinical Nursing Practice and Role Delineations* Bocian et al. (2020) USA	To provide examples for implementation of the Standards of Practice as they relate to the roles of: advanced practice nurses (APRN), registered nurses, licensed practical/vocational nurses (LPN/LVN), and nursing assistive personnel (NAP), in the gastroenterology role.	Descriptive study design	Patient assessment skills Medication administration Basic patient care Communication and teamwork Adherence to safety protocols Documentation and reporting
*Core Competencies Required for Gastroenterology Nursing Specialists in China* Ren et al. (2019) China	To define core competencies required for gastroenterology nursing specialists (GNS) in China.	Mixed‐method approach: literature review, interviews, and Delphi technique with 28 experts. Data analyzed with SPSS.	Identified 7 domains and 66 core competencies for GNS: Clinical practice Collaboration Leadership Education Ethical and legal practice Research
*Development of a Competency Framework Among Gastrointestinal Endoscopy Nurses, Ramkhamhaeng Hospital Group, Chiang Mai Province* Siriwat et al. (2020) Thailand	To develop a competency framework for gastrointestinal endoscopy nurses at Ramkhamhaeng Hospital Group, Chiang Mai Province.	Developmental study using interviews and literature review. Participants: 9 endoscopy nurses and 3 experts. Data analysis: content analysis and descriptive statistics.	Six competencies with 82 behavioral indicators: Care before gastrointestinal endoscopy Care during gastrointestinal endoscopy Care after gastrointestinal endoscopy Assistance to physicians in gastrointestinal endoscopy Record of documents related to gastrointestinal endoscopy Maintenance of gastrointestinal endoscopy tools and equipment.
*Standards of clinical nursing practice and role delineations in the gastroenterology setting* SGNA (2010) USA	To outline nursing standards and roles in gastroenterology.	Analysis of nursing responsibilities and collaboration standards.	Assessment Diagnosis Outcome identification Planning Implementation Evaluation Ethics Culturally competent practice Collaboration Leadership Education EBP and research Quality of practice Resource utilization
*Role delineations and professional practice competencies of gastrointestinal endoscopy nurses* Huang et al. (2021) China	To provide reference to identify the role delineations and practice standards of GI endoscopy nurse.	Literature review	Detection of small bowel lesions by capsule endoscopy Endoscopic placement of PEG (percutaneous endoscopic gastrostomy) Administration of sedation and anesthesia
*Importancia del rol de la enfermera en la calidad de la endoscopia digestiva: Enfoque hacia la práctica avanzada* Alouali Moussakhkhar et al. (2023) Spain	To understand the evolution and current situation of nurses in digestive endoscopy units, describe their role, and identify advanced practice competencies.	Literature review from MEDLINE, COCHRANE, Web of Science, DIALNET, CUIDEN, and Scopus; included 14 articles (less than 10 years) in English and Spanish.	Holistic perspective of caring Health education Expert clinical thinking Reflective practice Clinical practice based on the scientific method Expert in coaching Consultant Researcher Collaboration Clinical, professional, and systems leadership Decision‐making
*Nurse‐administered Propofol Sedation Training Curricula and Propofol administration in Digestive Endoscopy Procedures* Minciullo and Filomeno (2024) Italy	To verify requirements, types of training, and operating methods for nurse‐administered propofol sedation.	A scoping review of the literature following PRISMA‐ScR guidelines across four biomedical databases, focusing on studies published in the last 20 years.	Knowledge of sedation protocols Effective communication with the endoscopist Ability to manage complications during sedation Administration of sedation and anesthesia Monitoring vital signs
*Mapping training programs for endoscopy nurses in gastroenterology: a scoping review* Parrella et al. (2025) Italy	To map existing training programs for endoscopy nurses and examine their impact on clinical practice and patient care.	Scoping review using Arksey and O'Malley framework; analysis of studies from multiple databases (PubMed, Embase, Web of Science, Scopus, CINAHL).	Sedation administration Airway management Handling endoscopy equipment Interpreting capsule endoscopy images Communication Teamwork Crisis management
*Definition of Skills and Roles of the Digestive Endoscopy Nurse* Napolitano et al. (2025) Italy	Define the skills and roles of endoscopy nurses in Italy and propose progressive levels of training.	Working group with expert nurses and physicians using the Delphi method to outline training for different levels of endoscopy nurses.	Specialized knowledge and technical skills Nursing assessment and Intervention skills Health, safety, public hygiene, and cancer screening Control of infection in the endoscopic environment Knowledge and care of endoscopic equipment Professional and ethical practice Commitment to the development of professional practice

Competency domains were generated through inductive thematic synthesis. Extracted competency‐related statements from each included source were coded line‐by‐line. Similar codes were grouped into descriptive categories and iteratively consolidated into higher‐order domains based on conceptual similarity and recurrence across studies.

The final domains represent cross‐study convergence rather than predefined categories.

### 3.2 Core Competency Domains

Across sources, several core competency domains were consistently identified:

- Clinical assessment and patient management: encompassing pre‐, intra‐, and post‐procedural management, including assessment, preparation, monitoring, and recovery (Siriwat et al. 2020; SGNA 2014; Bocian et al. 2020; Napolitano et al. 2025; Gómez and Llach 2009).
- Procedural and interventional support competence: supporting diagnostic and therapeutic procedures, including specialized techniques such as capsule endoscopy and percutaneous endoscopic gastrostomy (SGNA 2010; Salvetto 2007).
- Equipment management and infection control: maintaining, disinfecting, and reprocessing equipment while adhering to strict hygiene protocols (ESGENA 2004; Beilenhoff et al. 2011; Napolitano et al. 2025; Salvetto 2007).
- Sedation and patient monitoring: particularly with the growing role of nurse‐administered sedation and associated training requirements (Minciullo and Filomeno 2024; Parrella et al. 2025; SGNA 2013).
- Documentation and quality assurance: accurate record‐keeping and adherence to institutional standards for safety and quality (SGNA 2013; Beilenhoff et al. 2011).

### 3.3 Advanced and Expanded Roles

Many documents described the evolution of endoscopy nursing into advanced practice roles. These included leadership, interprofessional collaboration, health education, coaching, research, and consultation (Alouali Moussakhkhar et al. 2023; Ren et al. 2019; Vuorinen et al. 2009). SGNA (2010) guidelines emphasized ethical practice, staff education, quality improvement, and clinical governance as essential components of professional growth. Asian frameworks, such as those by Ren et al. (2019), highlighted structured competency frameworks, Delphi‐consensus approaches, and expanded procedural responsibilities.

### 3.4 Regional Variability

Competency expectations varied by country. European and Italian sources prioritized technical proficiency, infection control, and organizational management (ESGENA 2004; Napolitano et al. 2025), while North American standards integrated cultural competence, evidence‐based practice, and comprehensive patient assessment (SGNA 2013, 2014; SGNA 2010). Asian contributions focused on systematic competency development, advanced procedural skills, and consensus‐driven role definitions (Siriwat et al. 2020; Ren et al. 2019).

### 3.5 Emerging Trends

Recent literature demonstrates a shift toward autonomous practice and advanced clinical responsibilities, including nurse‐administered sedation, crisis management, and leadership in quality assurance initiatives (Minciullo and Filomeno 2024; Parrella et al. 2025; Alouali Moussakhkhar et al. 2023). The integration of digital tools such as competency‐based e‐portfolios was identified as a key strategy for tracking, validating, and standardizing skills across settings (Parrella et al. 2025).

Overall, the findings illustrate a global transition of GI endoscopy nursing from a supportive technical role to a multidimensional, competency‐driven specialty incorporating clinical expertise, leadership, and continuous professional development.

## 4 Discussion

The findings from this review highlight the critical competencies required for nursing professionals in the field of gastrointestinal endoscopy, underscoring the complexities of their roles and the necessity for structured competency frameworks. The studies consistently identified a spectrum of competencies ranging from technical skills in procedural care to broader roles encompassing patient advocacy and ethical considerations.

The present synthesis highlights the evolution of gastrointestinal (GI) endoscopy nursing into a highly specialized, competency‐driven field. Across regions and study designs, there is strong agreement on the foundational domains of practice, while also revealing regional variations, emerging trends, and the expansion of professional roles.

For instance, studies from Europe, North America, and Asia included in this review explicitly reported both core technical competencies and additional responsibilities related to leadership, patient education, and quality improvement (Beilenhoff et al. 2011; SGNA 2010; Ren et al. 2019).

Consistently identified competency domains—clinical assessment and patient management, procedural and interventional support competence, equipment management, infection control, sedation, patient monitoring, documentation and quality assurance—reflect the multifaceted nature of endoscopy nursing. These findings align with previous studies (Calderwood et al. 2014), emphasizing that safe and effective endoscopy practice requires proficiency not only in technical skills but also in patient‐centered care, monitoring, and rigorous adherence to hygiene and safety protocols. The convergence of competencies across the included international studies suggests a growing consensus on core practice standards, which is critical for benchmarking and harmonizing training curricula (Moffat 2007).

The reviewed literature also underscores a notable shift from traditional supportive functions toward advanced practice roles encompassing leadership, interprofessional collaboration, health education, research, and clinical governance. This shift is evidenced in the included studies, which documented nurses' involvement in staff education, quality improvement initiatives, and structured competency development (SGNA 2010; Ren et al. 2019). These trends support the notion of endoscopy nursing as a distinct specialty requiring continuous professional growth (Viudez 2025).

While the core competencies are widely recognized, country differences remain evident and are consistent with previous research. European countries prioritize technical proficiency, infection control, and organizational management, whereas North American guidelines place greater emphasis on cultural competence, evidence‐based practice, and comprehensive patient assessment. Asian contributions highlight structured frameworks and advanced procedural competencies (Calderwood et al. 2014; Guarini et al. 2018; Nevin 2005). These observed differences in the included studies reflect variations in healthcare systems, educational structures, and regulatory environments. However, the emergence of harmonization efforts, such as standardized competency frameworks and Delphi‐consensus approaches, indicates a global movement toward aligning expectations, enhancing patient safety, and facilitating workforce mobility.

The ANOTE‐ANIGEA survey revealed substantial disparities in resources and practices across countries, particularly between Northern and Southern Italy. Moreover, the study highlighted a shortage of nursing coordinators, with over half lacking specialized postgraduate qualifications, and a significant portion of endoscopy nurses reporting inadequate theoretical training despite extensive practical experience (Guarini et al. 2018). These data directly support the interpretation that there is an urgent need for standardized training programs, enhanced infrastructure, and clear role delineation to ensure high‐quality and safe patient care in Italy.

The challenges identified in Italy, as reported in the included studies, reflect broader trends noted within the global literature analyzed in this review, showing an evolution of nursing roles toward advanced practice and competency‐based frameworks.

Consistent with our findings of advanced procedural responsibilities and skill gaps in Italy, Moffat (2007) demonstrates that structured academic and clinical training can enable nurses to safely perform endoscopies, illustrating how targeted education may address competency gaps identified in our review. Moffat (2007) describes a pioneering program enabling nurses from non‐medical backgrounds to perform endoscopies following structured academic and clinical training. This approach not only alleviates staffing constraints but also empowers nurses to take on advanced procedural responsibilities, contributing to reduced waiting times and optimized patient pathways. Such models demonstrate that investment in specialized education and skill development can yield tangible improvements in service delivery.

Similarly, in the United States, the evolution of endoscopy nursing has transitioned from a support function to a recognized specialty. Bauer and Sauer (2019) emphasize that modern endoscopy nurses must master complex sedation protocols, acute patient assessment, and post‐procedural care tailored to an increasingly elderly and comorbid population. Retaining skilled nurses in this demanding field requires more than initial training: it necessitates a supportive organizational culture, opportunities for mentorship, career advancement, and work‐life balance (Bauer and Sauer 2019). Shared governance models, continuing education, and recognition of professional contributions are central strategies to maintaining a committed and competent workforce. Several included studies emphasized the role of emerging technologies, such as digital competency tracking and e‐portfolios, in supporting skill validation and lifelong learning, providing concrete examples of how technological integration can enhance both accountability and professional development.

This literature review aligns also with recent literature pointing to increased autonomy, nurse‐administered sedation, crisis management, and leadership in quality initiatives as key trends shaping the future of GI endoscopy nursing (Embertson et al. 2020; Minciullo et al. 2022). The adoption of digital tools, including competency‐based e‐portfolios, provides innovative strategies for tracking, validating, and standardizing skills, thereby supporting lifelong learning and evidence‐based practice. These developments illustrate a broader transformation in nursing practice, where technological integration complements clinical expertise and fosters professional accountability (Bauer and Sauer 2019).

Several studies revealed that while awareness of EBP is prevalent among nurses, actual implementation in practice often lacks due to insufficient resources, confidence, and comprehensive training. To confront this, Munnelly et al. (2021) stated that targeted educational programs must be designed that emphasize practical application in real‐world settings alongside theoretical instruction.

Taken together, these findings illustrate that the role of the endoscopy nurse is no longer confined to technical assistance but encompasses clinical judgment, risk management, leadership, and patient advocacy. For Italy, aligning with international best practices will involve addressing structural deficiencies, formalizing specialist training pathways, and fostering a culture that values and supports professional growth. This integrated approach is essential to meet the rising demand for complex endoscopic procedures and to ensure that patient care remains safe, efficient, and evidence‐based.

Eventually, universities could strengthen the preparation of future nurse endoscopists by offering specialized training courses that go beyond the traditional nursing curriculum and focus on advanced competencies. Suggested courses might include gastrointestinal anatomy and physiology with emphasis on endoscopic correlation, advanced pharmacology and sedation management, diagnostic imaging interpretation, and evidence‐based practice in endoscopy. Practical modules in therapeutic techniques—such as polypectomy, hemostasis, and foreign body removal—should be complemented by simulation‐based training and supervised clinical placements in endoscopy units. Additional courses in communication, patient education, and interprofessional collaboration would help nurses develop the leadership and decision‐making skills required for autonomous practice. Finally, modules on research methods, quality improvement, and health policy could empower nurse endoscopists to contribute to innovation and service development in gastroenterology.

From a clinical standpoint, it is increasingly recognized that emerging technologies such as artificial intelligence (AI) and virtual reality (VR) simulation are reshaping training and competence acquisition in endoscopy, yet engagement with these tools has been limited in the current synthesis. *Virtual reality tools for gastrointestinal endoscopy training* is a recent systematic review demonstrating that VR‐based simulation enhances technical accuracy, reduces procedure time, and can decrease patient discomfort compared with traditional training approaches and that combining VR with conventional methods holds promise for improving outcomes in endoscopic skills acquisition (Dương and Soldera 2024). These findings suggest that continuous training in GI endoscopy can no longer rely solely on apprenticeship models or periodic courses; instead, structured integration of VR simulation and AI‐assisted feedback systems should be considered within competency frameworks. For nursing professionals, who increasingly participate in complex procedural care and quality assurance, technology‐enabled continuous learning can support safe upskilling alongside physicians, optimize psychomotor performance, and foster competency in advanced care delivery. Integrating such technologies explicitly into core competency frameworks would strengthen the field's responsiveness to rapid evolution in endoscopic techniques, devices, and quality metrics.

Future research should concentrate on developing a comprehensive and standardized repertoire of skills that can serve both as a framework for staff evaluation and as the foundation for competency‐based training programs tailored to nurse endoscopists. Studies should also explore nurses' perceptions of their roles, challenges, and learning needs, in order to design educational strategies that directly address gaps in knowledge and practice. Investigating the relationship between specific competencies and clinical outcomes would provide valuable evidence for refining training pathways and ensuring patient safety. Additionally, research should assess the effectiveness of different teaching methods—such as simulation, mentorship, and interprofessional learning—in enhancing both technical and non‐technical skills. Such efforts would not only support the continuous professional development of nurse endoscopists but also promote consistency, quality, and innovation in endoscopy services.

## 5 Strengths and Limitations

As a rapid review, this study streamlined certain systematic review processes, including exhaustive literature searches, which may have led to omission of relevant studies. The small number of included studies (*n* = 16) limits generalizability. Thematic synthesis may be influenced by reviewer interpretation, introducing potential bias in competency identification. Given the heterogeneity of sources, a single standardized risk‐of‐bias tool was not appropriate. For empirical studies (qualitative, mixed‐method, and developmental designs), methodological quality was assessed using Joanna Briggs Institute (JBI) tools, while guidelines, consensus statements, and position papers were not formally scored, as they provide professional standards rather than primary evidence. Despite these limitations, this rapid review offers preliminary insights to guide future research and workforce development in endoscopy nursing.

Future research should aim to explore with quantitative and qualitative methods on this topic, to capture a more comprehensive understanding of advanced nursing competencies in endoscopy. By adhering to these rigorous methods, this rapid review seeks to contribute valuable insights that inform educational strategies and workforce development for nurses in endoscopy, ultimately enhancing patient care and safety in this specialized clinical domain.

## 6 Conclusion

This review highlights the essential competencies and evolving roles of nurses in gastrointestinal endoscopy, demonstrating that practice increasingly demands not only technical proficiency but also clinical judgment, leadership, and patient advocacy.

Clinical assessment and patient management, procedural and interventional support competence, equipment management and infection control, sedation and patient monitoring, and documentation and quality assurance are the main core competencies found.

Findings suggest that structured competency frameworks, such as those developed by ESGENA and similar organizations, are critical for standardizing practice and ensuring patient safety. Evidence from the included studies supports implementing targeted educational programs that combine theoretical instruction, simulation‐based training, and supervised clinical placements to address identified skill gaps.

As the field increasingly integrates innovative technologies, including artificial intelligence, it is crucial for nursing professionals to engage with these advancements to enhance their practice and patient care. Given the small number and heterogeneity of studies included in this review, conclusions should be interpreted cautiously. Future research should evaluate the effectiveness of specific training interventions, the impact of advanced nursing roles on clinical outcomes, and strategies for integrating emerging technologies, including artificial intelligence, into endoscopy practice.

In conclusion, addressing the educational and professional needs of endoscopy nurses will not only empower them in their roles but also significantly contribute to the overall effectiveness of gastrointestinal endoscopy services, underpinning safer and higher‐quality patient experiences.

## 7 Relevance for Clinical Practice

The findings from this review underscore several key implications for nursing practice within the gastrointestinal endoscopy setting.

First, implementation of standardized curricula can facilitate the ongoing professional development of endoscopy nurses, ensuring they are well‐equipped to manage complex patient needs effectively.

Further, the role of nurses in monitoring patients during the post‐anesthetic recovery phase must be prioritized to ensure optimal patient safety (Sever and Hızlı 2023). The collaboration between nursing professionals and anesthesiology teams is critical in this context, as nurses must possess the knowledge and skills to assess potential complications during recovery.

The review also highlighted significant barriers to implementing evidence‐based practices in endoscopy units. This calls for healthcare organizations to foster a supportive environment conducive to continuous education and the application of best practices. Addressing gaps in institutional support can empower nurses to embrace evidence‐based guidelines and interventions, ultimately improving patient outcomes.

Moreover, studies indicating that nurse‐administered sedation is both safe and effective reinforce the need for training in sedation practices and adherence to guidelines (Dhawan 2020). Continued education regarding the appropriate use of sedation medications, monitoring techniques, and protocols to minimize risks during procedures is vital for maintaining high standards of patient care.

The implications also extend to ethical considerations. Providing ethical training and developing clear institutional guidelines can help nurses navigate these complexities and advocate effectively for patient rights.

Last, the incorporation of innovative technologies such as artificial intelligence (AI) in endoscopy signals a trend toward enhanced diagnostic accuracy and procedural efficiency (Ang and Carneiro 2021). It is crucial for nurses to engage in ongoing education regarding these technological advancements, ensuring they remain competent in utilizing new tools that can improve patient care.

## Author Contributions

Andrea Minciullo contributed to formal analysis, and writing – original draft preparation. Daniela Tartaglini contributed to methodology, supervision, and writing – review and editing. Lucia Filomeno contributed to investigation, data collection, and writing – original draft preparation. Riccardo Grande contributed to investigation and data collection. Dhurata Ivziku contributed to conceptualization, data curation, supervision, and writing – review and editing. All authors have read and agreed to the published version of the manuscript.

## Funding

This study was supported by a grant (reference number 3.25.2) received by the Centre of Excellence for Nursing Scholarship (CECRI), Order of Nurses of Rome, Italy. The funding organization had no impact on the design, implementation, or analysis of the research.

## Ethics Statement

Ethical approval was not required, as the study relied solely on publicly available data and did not include human or animal subjects. All data analyzed in this review were derived from published literature and publicly accessible documents. No individual‐level data were collected, accessed, or stored. Data management was limited to bibliographic records and extracted study characteristics, which were stored securely in password‐protected institutional files accessible only to the research team. Due to the rapid review design and inclusion of heterogeneous evidence types (e.g., guidelines and consensus documents), the protocol was not registered in PROSPERO, as this registry primarily accommodates systematic reviews of health outcomes.

## Conflicts of Interest

The authors declare no conflicts of interest.

## Data Availability

Data sharing not applicable to this article as no datasets were generated or analyzed during the current study.

## References

[nhs70328-bib-0001] Alouali Moussakhkhar, B. , J. Romero Xandre , R. Pérez Berbegal , M. Parrilla Carrasco , X. Font Lagarriga , and G. Casals Urquiza . 2023. “Importancia del rol de la Enfermera en la Calidad de la Endoscopia Digestiva: Enfoque Hacia la Práctica Avanzada.” www.aeeed.com. 10, 1, 4.

[nhs70328-bib-0049] Ang, T. L. , and G. Carneiro . 2021. “Artificial Intelligence in Gastrointestinal Endoscopy.” Journal of Gastroenterology & Hepatology 36, no. 1. 10.1111/jgh.15344 33448513

[nhs70328-bib-0002] Bauer, C. , and B. G. Sauer . 2019. “Growing and Retaining an Endoscopy Nurse.” Clinical Gastroenterology and Hepatology 17, no. 1: 5–7. 10.1016/j.cgh.2018.09.014. 30218705

[nhs70328-bib-0003] Beilenhoff, U. , C. S. Neumann , and M. Campbell Dortmann . 2011. “European Society of Gastroenterology and Endoscopy Nurses and Associates: European Core Curriculum for Endoscopy Nursing. 2007.” Zugriff, 17. http://www.esgena.org.

[nhs70328-bib-0004] Benner, P. 1984. “From Novice to Expert: Excellence and Power in Clinical Nursing Practice.” AJN, American Journal of Nursing 84: 1480.

[nhs70328-bib-0005] Bocian, S. , C. M. Loyola , M. Benitez‐Romero , et al. 2020. “Standards of Clinical Nursing Practice and Role Delineations in the Gastroenterology Setting.” Gastroenterology Nursing 43, no. 3: E129–E141. 10.1097/SGA.0000000000000535. 32487961

[nhs70328-bib-0006] Calderwood, A. H. , F. J. Chapman , J. Cohen , et al. 2014. “Guidelines for Safety in the Gastrointestinal Endoscopy Unit.” Gastrointestinal Endoscopy 79, no. 3: 363–372. 10.1016/j.gie.2013.12.015. 24485393 PMC3980655

[nhs70328-bib-0048] Dhawan, P. 2020. “COVID‐19 Pandemic Brings Gastrointestinal Endoscopy Practice to Its Knees—Financially!” Journal of Digestive Endoscopy 11, no. 1: 81–82. 40477068 10.1055/s-0040-1712548 PMC7356658

[nhs70328-bib-0007] Dunkley, I. , H. Griffiths , R. Follows , et al. 2019. “UK Consensus on Non‐Medical Staffing Required to Deliver Safe, Quality‐Assured Care for Adult Patients Undergoing Gastrointestinal Endoscopy.” Frontline Gastroenterology 10, no. 1: 24–34. 10.1136/flgastro-2017-100950. 30651954 PMC6319155

[nhs70328-bib-0008] Dương, T. Q. , and J. Soldera . 2024. “Virtual Reality Tools for Training in Gastrointestinal Endoscopy: A Systematic Review.” Artificial Intelligence in Gastrointestinal Endoscopy 5, no. 2: 92090. 10.37126/aige.v5.i2.92090.

[nhs70328-bib-0009] Embertson, A. , N. Ernst , J. Yoder , L. Monroe , and M. Hess . 2020. “Development of a Nurse‐Led Competency‐Based Program for Therapeutic Endoscopy.” Gastroenterology Nursing 43, no. 6: E217–E224. 10.1097/sga.0000000000000501. 33055546

[nhs70328-bib-0010] European Society of Gastroenterology and Endoscopy Nurses and Associates (E.S.G.E.N.A.) . 2004. “European Job Profile for Endoscopy Nurses: August 2004.” Endoscopy 36, no. 11: 1025–1030. 10.1055/s-2004-825963. 15520926

[nhs70328-bib-0011] Fang, L. , B. Wu , P. Wang , L. Chen , and Y. Xu . 2025. “Development and Validation of a Competency Evaluation Index System for Nurse Endoscopists With Different Stages Performing Endoscopy Nursing in China: A Modified Delphi Study.” Nurse Education Today 144: 106411. 10.1016/j.nedt.2024.106411. 39305722

[nhs70328-bib-0012] Field, D. E. 2004. “Moving From Novice to Expert—The Value of Learning in Clinical Practice: A Literature Review.” Nurse Education Today 24, no. 7: 560–565. 10.1016/j.nedt.2004.07.009. 15465172

[nhs70328-bib-0013] Garritty, C. , C. Hamel , M. Trivella , et al. 2024. “Updated Recommendations for the Cochrane Rapid Review Methods Guidance for Rapid Reviews of Effectiveness.” BMJ (Clinical Research ed.) 384: e076335. 10.1136/bmj-2023-076335. 38320771

[nhs70328-bib-0014] Gómez, M. , and J. Llach . 2009. “Papel de la Enfermera en la Endoscopia Digestiva.” Gastroenterology 32: 44–47. 10.1016/j.gastrohep.2008.02.002. 19174099

[nhs70328-bib-0015] Guarini, A. , E. Rossetti , P. Simonelli , et al. 2018. “L'infermiere Nelle Endoscopie Digestive in Italia: Una Survey Nazionale di Anote‐Anigea.” L'infermiere 53, no. 1: 22–28.

[nhs70328-bib-0016] Huang, C. , Y. Liu , and H. Lu . 2021. “Role Delineations and Professional Practice Competencies of Gastrointestinal Endoscopy Nurses.” Chinese Journal of Gastrointestinal Endoscopy (Electronic Edition) 8, no. 2: 89. 10.3877/cma.j.issn.2095-7157.2021.02.010.

[nhs70328-bib-0017] King, V. J. , A. Stevens , B. Nussbaumer‐Streit , C. Kamel , and C. Garritty . 2022. “Paper 2: Performing Rapid Reviews.” Systematic Reviews 11, no. 1: 151. 10.1186/s13643-022-02011-5. 35906677 PMC9338520

[nhs70328-bib-0018] Li, C. , X. Du , X. Tian , J. Yao , and S. Tian . 2024. “A Practical Index System Forevaluating the Core Competence of Specialized Nurses in Digestive Endoscopy.” 10.21203/rs.3.rs-4681427/v1.

[nhs70328-bib-0019] McClelland, D. C. 1976. A Guide to Job Competency Assessment, 178. McBer.

[nhs70328-bib-0020] Mijumbi‐Deve, R. M. , I. Kawooya , E. Kayongo , et al. 2022. “Paper 1: Demand‐Driven Rapid Reviews for Health Policy and Systems Decision‐Making: Lessons From Lebanon, Ethiopia, and South Africa on Researchers and Policymakers' Experiences.” Systematic Reviews 11, no. 1: 154. 10.1186/s13643-022-02021-3. 35907879 PMC9338611

[nhs70328-bib-0021] Minciullo, A. , B. Colombo , D. Tartaglini , L. Filomeno , and F. Maria Di Matteo . 2022. “Inspection of a Duodenoscope Working Channel After a Double Positive Sampling: Case Report and Literature Review.” Gastrointestinal Nursing 20, no. Sup1: S26–S29.

[nhs70328-bib-0022] Minciullo, A. , and L. Filomeno . 2024. “Nurse‐Administered Propofol Sedation Training Curricula and Propofol Administration in Digestive Endoscopy Procedures: A Scoping Review of the Literature.” Gastroenterology Nursing: The Official Journal of the Society of Gastroenterology Nurses and Associates 47, no. 1: 33–40. 10.1097/SGA.0000000000000780. 37937982

[nhs70328-bib-0046] Moffat, P. 2007. “Expansion of the Nurse Role.” Gastrointestinal Nursing 5, no. 10: 3.

[nhs70328-bib-0023] Moore, G. , S. Redman , S. Rudge , and A. Haynes . 2018. “Do Policy‐Makers Find Commissioned Rapid Reviews Useful?” Health Research Policy and Systems 16, no. 1: 17. 10.1186/s12961-018-0293-1. 29482643 PMC5828139

[nhs70328-bib-0024] Munnelly, S. , V. Howard , V. Hall , J. Richardson , and M. Kirkbride . 2021. “Knowledge and Education to Inform Evidence‐Based Practice in Gastrointestinal Nursing: A Scoping Review.” Gastrointestinal Nursing 19: 36–45. 10.12968/gasn.2021.19.6.36.

[nhs70328-bib-0025] Napolitano, D. , M. Candela , M. Gaggiotti , et al. 2025. “Definition of Skills and Roles of the Digestive Endoscopy Nurse: Italian Consensus of ANOTE‐ANIGEA.” Gastroenterology Nursing 48: 361–371. 10.1097/SGA.0000000000000887. 40556058 PMC12459133

[nhs70328-bib-0026] Nevin, C. B. 2005. “Mini Doctors or Advanced Nurse Practitioners?: Irish Endoscopy Nurses' Perceptions Regarding the Development of Advanced Practice in Endoscopy.” Gastroenterology Nursing: The Official Journal of the Society of Gastroenterology Nurses and Associates 28, no. 4: 285–290. 10.1097/00001610-200507000-00002. 16189403

[nhs70328-bib-0027] Notarnicola, I. , D. Ivziku , D. Tartaglini , et al. 2023. “Self‐Perceived Clinical Competence of Nurses in Different Working Experiences: A Cross‐Sectional Study.” Healthcare (Basel) 11, no. 21: 2808. 37957953 10.3390/healthcare11212808 PMC10648505

[nhs70328-bib-0028] Page, M. J. , J. E. McKenzie , P. M. Bossuyt , et al. 2021. “The PRISMA 2020 Statement: An Updated Guideline for Reporting Systematic Reviews.” BMJ 372: n71. 10.1136/bmj.n71. 33782057 PMC8005924

[nhs70328-bib-0029] Parrella, A. , D. Rusconi , A. Povoli , et al. 2025. “Mapping Training Programs for Endoscopy Nurses in Gastroenterology: A Scoping Review.” European Journal of Gastroenterology & Hepatology 37, no. 6: 702–709. 10.1097/MEG.0000000000002951. 40207485

[nhs70328-bib-0030] Prahalad, C. K. , and G. Hamel . 2007. “Strategy as a Field of Study: Why Search for a New Paradigm?” Strategic Management Journal 15: 5–16. 10.1002/smj.4250151002.

[nhs70328-bib-0031] Ren, H. , C. Liu , R. Wang , et al. 2019. “Core Competencies Required for Gastroenterology Nursing Specialists in China.” Gastroenterology Nursing: The Official Journal of the Society of Gastroenterology Nurses and Associates 42, no. 2: 169–178. 10.1097/SGA.0000000000000392. 30946304

[nhs70328-bib-0032] Salvetto, M. 2007. “Ruolo Dell'infermiere in Ecoendoscopia.” Minerva Medica 98, no. 4: 269–270. 17921937

[nhs70328-bib-0033] Schoefl, R. 2023. “Endoscopic Retrograde Cholangio Pancreatography and Endoscopic Ultrasound: The Pilot Is Only as Good as the Co‐Pilot—What Do Nurses Need to Know?” Gastrointestinal Nursing 21: 20–24. 10.12968/gasn.2023.21.7.20.

[nhs70328-bib-0047] Sever, F. , and Ş. Hızlı . 2023. “Awareness of Endoscopy Nurses About Anesthesia Management in the Pediatric Gastrointestinal Endoscopy Unit; A Survey Study.” Türkiye Çocuk Hastalıkları Dergisi 17, no. 5: 412–417.

[nhs70328-bib-0034] Siau, K. , J. T. Green , N. D. Hawkes , et al. 2019. “Impact of the Joint Advisory Group on Gastrointestinal Endoscopy (JAG) on Endoscopy Services in the UK and Beyond.” Frontline Gastroenterology 10, no. 2: 93–106. 10.1136/flgastro-2018-100969. 31210174 PMC6540274

[nhs70328-bib-0035] Siriwat, S. , K. Abhicharttibutra , and T. Supamanee . 2020. “Development of a Competency Framework Among Gastrointestinal Endoscopy Nurses.” Nursing Journal CMU 47, no. 2: 333–344.

[nhs70328-bib-0038] Society of Gastroenterology Nurses and Associates (SGNA) . 2010. Position Statement: Manipulation of Gastroenterology Endoscopes During Endoscopic Procedures. SGNA. 10.1097/sga.0b013e3181d92b44. 1878391

[nhs70328-bib-0036] Society of Gastroenterology Nurses and Associates (SGNA) . 2013. Guidelines for Nursing Documentation in Gastrointestinal Endoscopy. SGNA. 10382416

[nhs70328-bib-0037] Society of Gastroenterology Nurses and Associates (SGNA) . 2014. Standards of Clinical Nursing Practice and Role Delineations. Society of Gastroenterology Nurses and Associates, SGNA. 10.1097/SGA.0000000000000535 32487961

[nhs70328-bib-0039] Sprout, J. 2000. “Nurse Endoscopist Training: The Next Step.” Gastroenterology Nursing 23, no. 3: 111–115. 11235441 10.1097/00001610-200005000-00004

[nhs70328-bib-0040] Spry, C. , and M. Mierzwinski‐Urban . 2018. “The Impact of the Peer Review of Literature Search Strategies in Support of Rapid Review Reports.” Research Synthesis Methods 9, no. 4: 521–526. 10.1002/jrsm.1330. 30408843

[nhs70328-bib-0041] Tricco, A. C. , S. E. Straus , A. Ghaffar , and E. V. Langlois . 2022. “Rapid Reviews for Health Policy and Systems Decision‐Making: More Important Than Ever Before.” Systematic Reviews 11, no. 1: 153. 10.1186/s13643-022-01887-7. 35906637 PMC9338614

[nhs70328-bib-0042] Viudez, G. C. 2025. “La Figura del Profesional de Enfermería de Práctica Avanzada en las Unidades de Endoscopia Digestiva.” Gastroenterología y Hepatología 48, no. 3: 502260. 10.1016/j.gastrohep.2024.502260. 39322065

[nhs70328-bib-0043] Vuorinen, R. , E. Heino , and R. Meretoja . 2009. “Endoscopy Nurse as a Patient Care Coordinator: The Expanded Role of the Competent Nurse in the Gastroenterology Setting.” Gastroenterology Nursing 32, no. 6: 410–413. 10.1097/SGA.0b013e3181c53799. 20010234

[nhs70328-bib-0044] Xu, C. , K. Ju , L. Lin , et al. 2022. “Rapid Evidence Synthesis Approach for Limits on the Search Date: How Rapid Could It Be?” Research Synthesis Methods 13, no. 1: 68–76. 10.1002/jrsm.1525. 34523791

[nhs70328-bib-0045] Yu, S. , and Y. S. Roh . 2018. “Needs Assessment Survey for Simulation‐Based Training for Gastrointestinal Endoscopy Nurses.” Nursing & Health Sciences 20, no. 2: 247–254. 10.1111/nhs.12412. 29377577

